# The Beneficial and Harmful Effects of Perioperative Clonidine: Protocol for a Systematic Review With Meta‐Analysis

**DOI:** 10.1111/aas.70101

**Published:** 2025-07-15

**Authors:** Stine Birkebæk, Caroline Barkholt Kamp, Nicholas Papadomanolakis‐Pakis, Janus Christian Jakobsen, Lone Nikolajsen, Peter Gaarsdal Uhrbrand

**Affiliations:** ^1^ Department of Clinical Medicine Aarhus University Aarhus Denmark; ^2^ Copenhagen Trial Unit, Centre for Clinical Intervention Research The Capital Region, Copenhagen University Hospital ‐ Rigshospitalet Copenhagen Denmark; ^3^ Department of Regional Health Research The Faculty of Health Sciences, University of Southern Denmark Odense Denmark; ^4^ Department of Anaesthesiology and Intensive Care Aarhus University Hospital Aarhus Denmark

## Abstract

**Background:**

Moderate to severe postoperative pain is frequent in patients undergoing surgery and is associated with increased morbidity and prolonged hospitalisation. Therefore, sufficient pain management should be prioritised. Clonidine, an alpha‐2 agonist with analgesic properties, may represent a relevant option in postoperative pain management. While clonidine is commonly used in a clinical setting, the quality of evidence supporting its perioperative use is limited by small sample sizes, heterogeneous study designs, and variations in administration routes. This systematic review aims to evaluate the benefits and harms of perioperative clonidine in postoperative pain management.

**Methods:**

We will conduct a systematic review of randomised clinical trials assessing the effects of perioperative clonidine on postoperative pain intensity, adverse effects, and postoperative opioid consumption. This review will include trials using perioperative systemic (oral, intravenous, transdermal, or intramuscular) clonidine and trials using placebo or no intervention as the control intervention. We will systematically search Medline, Embase, and the Cochrane Central Register of controlled trials for relevant literature. The recommendations by the Cochrane Collaboration and the Preferred Reporting Items for Systematic Reviews and Meta‐Analysis Protocols (PRISMA‐P) are followed for this protocol, and the PRISMA guidelines will be followed in the actual review. The certainty of evidence will be assessed using the Grading of Recommendations Assessment, Development, and Evaluation (GRADE) approach.

**Outcomes:**

The primary outcomes are postoperative pain intensity and serious adverse events. Secondary outcomes include postoperative opioid consumption, quality of life, and non‐serious adverse events.

**Discussion:**

Although clonidine is used in a clinical setting to manage postoperative pain, the benefits and harms of this intervention remain unclear.

**Conclusion:**

This systematic review will provide valuable information on the efficacy and safety of perioperative clonidine in postoperative pain management.

## 1 Introduction

### 1.1 Postoperative Pain

Postoperative pain typically involves both nociceptive and neuropathic components. Nociceptive pain arises from both direct activation of neural pathways due to tissue damage and the release of inflammatory mediators from the surgical incision. Neuropathic pain arises from nerve injury [1]. Therefore, effective postoperative pain management requires a multimodal approach targeting both mechanisms. A considerable proportion of approximately 20% of surgical patients experience moderate to severe postoperative pain [2]. Inadequate pain control is associated with reduced patient satisfaction, prolonged hospitalisation, and an increased risk of developing persistent pain [3, 4, 5]. Especially, persistent pain represents a major international health issue [6]. Persistent pain is linked to diminished quality of life and may lead to psychosocial distress, insomnia, and depressive symptoms [7, 8].

### 1.2 Multimodal Pain Management

Today, multimodal pain management is widely used to relieve postoperative pain. Despite this, opioids remain the mainstay for moderate to severe pain due to their effectiveness in short‐term pain control. However, opioids have several undesirable adverse effects for patients, including nausea, vomiting, sedation, constipation, and the risk of long‐term dependence [9]. Thus, there is an urgent need to identify alternative analgesics that may enable a reduction in opioid use and associated complications to ultimately improve postoperative pain management.

### 1.3 Description of the Intervention

Clonidine is an alpha‐2 adrenergic agonist with analgesic properties and may be a useful adjunct in multimodal pain management. Clonidine exerts its effects by binding to alpha‐2 adrenergic receptors in the central nervous system, leading to reduced sympathetic outflow. This diminishes sympathetic transmission to the heart, kidneys, and peripheral vasculature, resulting in decreased arterial blood pressure and heart rate [10]. While the exact mechanism of analgesia is not fully understood, it is believed to involve the activation of alpha‐2 adrenergic receptors at both spinal and supraspinal levels [11, 12]. Clonidine is rapidly and almost completely absorbed, with maximum plasma concentration occurring 2 h after oral administration and 10 min after intravenous administration [10, 12]. Excretion occurs primarily through the kidneys, with an elimination half‐life ranging from 6 to 23 h [10, 12, 13, 14]. Clonidine can be administered via oral, intravenous, intramuscular, transdermal, epidural, intrathecal, and perineural (in peripheral nerve blocks) routes. Common adverse effects include dizziness, sedation, nausea, and constipation. Additional adverse effects may include sinus bradycardia, atrioventricular block, bradyarrhythmia, and hypotension [10]. Clonidine was initially marketed as an antihypertensive agent and subsequently adopted in anaesthesia for analgesia, sedation, shivering, and reduced anaesthetic requirements. Today, clonidine is used to treat shivering, postoperative pain, and opioid withdrawal symptoms [10, 15].

### 1.4 Relevance of This Review

We identified three previous systematic reviews with meta‐analyses of trials evaluating the role of alpha‐2 agonists in anaesthesia [15, 16, 17]. The most recent review, published in 2020, examined the effects of preoperative systemic alpha‐2 agonists (clonidine and dexmedetomidine) on postoperative pain and opioid consumption [16]. This review found reductions in both postoperative pain intensity and opioid consumption; however, these findings were constrained due to heterogeneity and potential small‐study bias. A systematic review from 2017 evaluated the potential indications of perioperative systemic clonidine in anaesthesia and found similar effects on postoperative pain [15]. In addition, the authors found a reduced risk of postoperative nausea and vomiting, postoperative shivering, and improved haemodynamic stability, without any influence on renal or cardiac outcomes. A systematic review from 2012 also investigated whether perioperative systemic alpha‐2 agonists (clonidine and dexmedetomidine) decreased postoperative pain and opioid consumption [17]. This study found reductions in postoperative opioid consumption, pain intensity, and nausea, but data on adverse events were limited. None systematically assessed adverse events. Additionally, none of the reviews employed Trial Sequential Analysis to evaluate imprecision or reduce the risk of random errors, nor did they assess the certainty of evidence using the GRADE (Grading of Recommendations Assessment, Development, and Evaluation) approach. Finally, no protocols were published for any of the included reviews.

Considering the increased attention on clonidine in the last decade, the high degree of heterogeneity and lack of certainty of evidence from previous reviews, there is a need for an updated systematic review to assess the benefits and harms of perioperative clonidine for postoperative pain management. We will minimise the risk of random error by using Trial Sequential Analysis, conduct subgroup analyses to assess heterogeneity, assess the risk of bias using the revised Cochrane Risk of Bias Tool for randomised trials (RoB 2), and evaluate the certainty of the evidence using GRADE [18, 19].

### 1.5 Objectives

The purpose of this systematic review is to assess the benefits and harms of perioperative use of clonidine in postoperative pain management.

## 2 Methods

This protocol has been developed based on the recommendations of the Cochrane Collaboration [18] and the Preferred Reporting Items for Systematic Reviews and Meta‐Analysis Protocols (PRISMA‐P) [19, 20] and has been registered on the Prospective Register of Systematic Reviews (PROSPERO) under the following registration number: CRD42023395510.

### 2.1 Criteria for Considering Studies for This Review

#### 2.1.1 Type of Studies

We will include randomised clinical trials, irrespective of trial design, setting, publication year, and language.

#### 2.1.2 Types of Participants

All adult patients (18 years or older) undergoing surgery, regardless of sex or comorbidities.

Trials involving paediatric patients will be excluded due to differences in pharmacokinetics, pharmacodynamics, and dose compared to adults. Furthermore, clonidine is frequently used for other indications in children, such as postoperative agitation. Therefore, it is not appropriate to combine paediatric and adult trials in a meta‐analysis.

#### 2.1.3 Experimental Intervention

Trials will be included if the intervention involves perioperative (immediate preoperative, intraoperative, or immediate postoperative) systemic administration of clonidine via oral, intravenous, transdermal, or intramuscular routes, regardless of the dose of clonidine administered.

#### 2.1.4 Control Intervention

As control interventions, we will accept no intervention or placebo.

### 2.2 Outcomes

#### 2.2.1 Primary Outcome

1. Postoperative pain intensity at rest, assessed on a visual analogue scale (VAS) or numerical rating scale (NRS), at the time point reported closest to 2 postoperative hours. We will also assess the pain intensity at rest at the time point closest to 12 postoperative hours.
2. Proportion of patients with serious adverse events (SAE) at maximum follow‐up
  ○ We will use the International Council for Harmonisation's Good Clinical Practice (ICH‐GCP) definition for a serious adverse event [21]. This is defined as any untoward medical occurrence that resulted in death, was life‐threatening, or led to significant disability, need for circulatory support, or prolonged hospitalisation. If previous studies did not use the ICH‐GCP definition or use the term serious adverse event, then we will also include the data if an event clearly fulfils the ICH‐GCP definition for a serious adverse event. We will secondly assess each type of serious adverse event separately.

#### 2.2.2 Secondary Outcomes

1. Postoperative opioid consumption (morphine equivalents) at maximum follow‐up within 24 h
2. Quality of life measured on any validated scale at maximum follow‐up
3. Proportion of patients with non‐serious adverse events at maximum follow‐up. Each adverse event will be analysed separately.

### 2.3 Information Sources

A systematic search will be conducted in three relevant medical databases, including the Medical Literature Analysis and Retrieval System Online (MEDLINE), Excerpta Medica Database (EMBASE), and the Cochrane Central Register of Controlled Trials (CENTRAL). There will be no date restrictions. The search strategy was informed by the randomised controlled trial design search filters by Haynes et al. and developed in collaboration with an experienced information specialist [22]. The reference lists of the included articles will be checked for relevant forward citations. The search strategy is included in Data S1.

#### 2.3.1 Selection Process

Two reviewers will independently screen titles and abstracts identified through the systematic search using Covidence systematic review software (Covidence, Veritas Health Innovation, Melbourne, Australia; available at www.covidence.org). Potentially relevant studies will then be assessed through full‐text reading by the same two reviewers. Any disagreement regarding inclusion or exclusion will be resolved with a third reviewer. The systematic review will include a PRISMA flow diagram showing the reasons for excluding full‐text articles, as well as the number of studies remaining after each stage of the selection process.

### 2.4 Data Extraction and Management

Two reviewers will independently extract data on predefined variables using the Covidence software. Any discrepancy regarding the extracted data will be identified and resolved through discussion. Duplicate publications and publications from the same main trial will be assessed to evaluate all available data simultaneously and maximise data extraction. We will contact authors to obtain additional data that might not have been reported sufficiently or at all in the published article. We will provide a narrative synthesis of the findings from the included studies. Results will be reported as presented in the original studies.

The following data will be extracted from each included trial:

– Study information
  ○ Title
  ○ First author
  ○ Year of publication
  ○ Geographical location (country, continent)
  ○ Trial design (placebo)
  ○ Inclusion and exclusion criteria
– Patient selection
  ○ Number of screened patients
  ○ Number of randomised patients
  ○ Number of analysed patients (sample size)
  ○ Number of patients in the clonidine group
  ○ Number of patients in the placebo group
  ○ Demographic characteristics
    - Age (mean/median)
    - Sex (proportion of females/males)
    - American Society of Anesthesiologists (ASA) physical status classification (proportion in each ASA classification)
  ○ Type of surgery
  ○ Type of anaesthesia
– Outcomes
  ○ Outcomes as defined above and by data availability

Depending on the data provided in the reviewed studies, specific data elements may be omitted, or additional data elements may be added for extraction.

#### 2.4.1 Variables Related to Perioperative Clonidine

– Route of administration (oral, intravenous, transdermal, or intramuscular)
– Dose of clonidine
– Timing of administration of clonidine (preoperative, intraoperative, postoperative)
– Loading dose with or without continuous infusion
– Duration of treatment

#### 2.4.2 Variables Related to Perioperative Placebo

– Route of administration
– Timing of administration of placebo (preoperative, intraoperative, postoperative)
– Duration of treatment

### 2.5 Risk of Bias Assessment

Two reviewers will independently assess the risk of bias in each included study using version 2 of the revised Cochrane Risk of Bias Tool for randomised trials (RoB 2) with respect to the effect of assignment to intervention [23].

The following domains will be evaluated:

1. Risk of bias arising from the randomization process
2. Risk of bias due to deviations from the intended interventions (effect of assignment to intervention)
3. Risk of bias due to missing outcome data
4. Risk of bias in measurement of the outcome
5. Risk of bias in selection of the reported result
6. Overall risk of bias
7. Risk of bias in each domain will be judged as low, high, or some concerns.

Only if all components are rated as having a low risk of bias, the overall classification will be low risk of bias. Disagreements will be resolved with a third reviewer. The main conclusion of this review will be based on results from studies judged to be at low risk of bias.

### 2.6 Data Synthesis

#### 2.6.1 Continuous Outcomes

For continuous outcomes, data will be extracted as means with standard deviations. Mean differences will be calculated at the study level and, where appropriate, pooled to calculate a weighted mean difference (WMD) with a 95% confidence interval (CI).

#### 2.6.2 Dichotomous Outcomes

For dichotomous outcomes, data will be extracted as the number of patients or events per group. Risk ratios (RR) with 95% CI will be calculated at the study level and pooled whenever possible.

#### 2.6.3 Missing Data

We will use intention‐to‐treat data if provided by the trialists. Study authors will be contacted to acquire relevant missing data. In cases of missing data or if medians rather than means are reported, SDs will be estimated according to formulas described by Cochrane [24] and means imputed based on the sample size, median, and IQR as described by Wan et al. [25]. Missing data will not be imputed in the primary analysis, but imputations will be used in our sensitivity analysis.

### 2.7 Statistical Methods

#### 2.7.1 Assessment of Heterogeneity

Data will be synthesised in accordance with the PRISMA guidelines. Studies will be assessed for clinical, methodological, and statistical heterogeneity. Forrest plots will be used to visually assess heterogeneity, while the proportion of total variability due to between‐study variance will be estimated using the *I* ^2^ test statistics with 95% CIs and chi^2^‐test [26, 27]. Ultimately, a meta‐analysis may not be considered in the event of high heterogeneity (clinical, methodological, or statistical). Instead, a narrative synthesis will be performed.

#### 2.7.2 Assessment of Publication Bias

Potential publication bias will be assessed using Egger's test and visually represented with funnel plots if 10 or more trials are included in a meta‐analysis. Alternatively, small study effects will be examined with forest plots to assess whether small RCTs are more likely to report larger treatment effects, compared with larger RCTs [28, 29, 30].

#### 2.7.3 Meta‐Analysis

We will carry out a meta‐analysis according to international recommendations stated in the Cochrane Handbook for Systematic Reviews of Intervention [24]. We will use the statistical software Stata to analyse data. We will assess the effects of our interventions with both a random‐effect meta‐analysis and a fixed‐effect meta‐analysis. Analyses and conclusions will be based on our primary outcomes. We will assess a total of two primary outcomes and consider a *p* value of 0.03 (adjustment per recommendations by Jakobsen et al.) or less as the threshold for statistical significance [31]. We will investigate possible heterogeneity through subgroup analyses. Subgroup analyses based on timing, dose, and route of intervention administration and type of surgery will be conducted if there is an adequate number of studies available.

#### 2.7.4 Trial Sequential Analysis

The risk of random errors due to sparse data and repetitive testing of data is a risk when performing a meta‐analysis. To control the risk of Type I and Type II errors, we will perform Trial Sequential Analysis [32].

### 2.8 Summary of Findings

We will create a summary of findings table for the primary and secondary outcomes. We will first present results based on trials assessed as having a low risk of bias, followed by results from all included trials.

Two reviewers will independently evaluate the overall certainty of evidence using the GRADE approach [33]. The reviewers will resolve disagreements by discussion with a third reviewer to make a final decision.

## 3 Discussion

Multimodal pain management is a cornerstone in relieving postoperative pain. Despite its widespread implementation, a substantial proportion of patients continue to suffer from moderate to severe postoperative pain. Although several reviews have examined the use of alpha‐2 agonists in anaesthesia, no previous systematic review has applied Trial Sequential Analysis to assess the risk of random errors, evaluated the quality of the evidence using the GRADE approach, or included trials published in the last 10 years.

This protocol outlines a systematic review designed to evaluate the beneficial and harmful effects of perioperative clonidine for postoperative pain management.

This protocol has several strengths. We have based the protocol on the PRISMA‐P checklist [20], and follow the methodological guidance provided in the Cochrane Handbook for Systematic Reviews of Interventions [24]. We will use RoB 2, Trial Sequential Analysis, and GRADE to assess the risk of systematic and random errors. Our protocol also has several limitations. We will pool data from all trials regarding the route of administration, timing of administration, and dose of clonidine, which will give rise to statistical and clinical heterogeneity. Additionally, we include patients undergoing various surgical procedures, which further increases clinical heterogeneity. Another limitation is that only randomised clinical trials will be included, potentially excluding relevant observational data. If heterogeneity is detected, we will consider whether a meta‐analysis is appropriate or whether a narrative synthesis is more applicable.

This protocol outlines a systematic review designed to evaluate the beneficial and harmful effects of perioperative clonidine for postoperative pain management.

## Author Contributions

S.B., P.G.U., and L.N. initiated this systematic review. S.B., P.G.U., C.B.K., N.P.P., J.C.J., and L.N. contributed to the conceptualisation, methodology, review, and editing. All authors have read and approved the final manuscript. The collaboration was conducted via the CEPRA network.

## Disclosure

In the event of protocol amendments, the data of each amendment will be accompanied by a description of the change and rationale. According to the PRISMA‐P guidelines, our systematic review has been registered on PROSPERO (CRD42023395510).

## Conflicts of Interest

The authors declare no conflicts of interest.

## Supporting information


**Data S1.** Supporting Information.

## Data Availability

The data that support the findings of this study are available on request from the corresponding author. The data are not publicly available due to privacy or ethical restrictions.

## References

[1] E. M. Pogatzki‐Zahn , D. Segelcke , and S. A. Schug , “Postoperative Pain‐From Mechanisms to Treatment,” Pain Reports 2, no. 2 (2017): e588. 29392204 10.1097/PR9.0000000000000588 PMC5770176

[2] A. M. Rasmussen , M. H. Toft , H. N. Awada , et al., “Waking up in Pain: A Prospective Unselected Cohort Study of Pain in 3702 Patients Immediately After Surgery in the Danish Realm,” Regional Anesthesia and Pain Medicine 46, no. 11 (2021): 948–953. 34408068 10.1136/rapm-2021-102583

[3] J. Katz and Z. Seltzer , “Transition From Acute to Chronic Postsurgical Pain: Risk Factors and Protective Factors,” Expert Review of Neurotherapeutics 9, no. 5 (2009): 723–744. 19402781 10.1586/ern.09.20

[4] H. Kehlet , T. S. Jensen , and C. J. Woolf , “Persistent Postsurgical Pain: Risk Factors and Prevention,” Lancet 367, no. 9522 (2006): 1618–1625. 16698416 10.1016/S0140-6736(06)68700-X

[5] P. Lavand'homme , “Transition From Acute to Chronic Pain After Surgery,” Pain 158, no. Suppl 1 (2017): S50–S54. 28134653 10.1097/j.pain.0000000000000809

[6] O. Gureje , M. Von Korff , G. E. Simon , and R. Gater , “Persistent Pain and Well‐Being: A World Health Organization Study in Primary Care,” Journal of the American Medical Association 280, no. 2 (1998): 147–151. 9669787 10.1001/jama.280.2.147

[7] S. N. Davison and G. S. Jhangri , “Impact of Pain and Symptom Burden on the Health‐Related Quality of Life of Hemodialysis Patients,” Journal of Pain and Symptom Management 39, no. 3 (2010): 477–485. 20303025 10.1016/j.jpainsymman.2009.08.008

[8] P. L. Kimmel , S. L. Emont , J. M. Newmann , H. Danko , and A. H. Moss , “ESRD Patient Quality of Life: Symptoms, Spiritual Beliefs, Psychosocial Factors, and Ethnicity,” American Journal of Kidney Diseases 42, no. 4 (2003): 713–721. 14520621 10.1016/s0272-6386(03)00907-7

[9] M. D. Neuman , B. T. Bateman , and H. Wunsch , “Inappropriate Opioid Prescription After Surgery,” Lancet 393, no. 10180 (2019): 1547–1557. 30983590 10.1016/S0140-6736(19)30428-3 PMC6556783

[10] S. Amna , T. Øhlenschlaeger , E. A. Saedder , J. V. Sigaard , and T. K. Bergmann , “Review of Clinical Pharmacokinetics and Pharmacodynamics of Clonidine as an Adjunct to Opioids in Palliative Care,” Basic & Clinical Pharmacology & Toxicology 134, no. 4 (2024): 485–497. 38275186 10.1111/bcpt.13979

[11] T. L. Yaksh , “Pharmacology of Spinal Adrenergic Systems Which Modulate Spinal Nociceptive Processing,” Pharmacology, Biochemistry, and Behavior 22, no. 5 (1985): 845–858. 2861606 10.1016/0091-3057(85)90537-4

[12] S. Jamadarkhana and S. Gopal , “Clonidine in Adults as a Sedative Agent in the Intensive Care Unit,” Journal of Anaesthesiology Clinical Pharmacology 26, no. 4 (2010): 439–445. 21547166 PMC3087273

[13] M. F. De Kock , G. Pichon , and J. L. Scholtes , “Intraoperative Clonidine Enhances Postoperative Morphine Patient‐Controlled Analgesia,” Canadian Journal of Anesthesia 39, no. 6 (1992): 537–544. 1643675 10.1007/BF03008314

[14] J. M. Bernard , J. L. Hommeril , N. Passuti , and M. Pinaud , “Postoperative Analgesia by Intravenous Clonidine,” Anesthesiology 75, no. 4 (1991): 577–582. 1928767 10.1097/00000542-199110000-00006

[15] M. C. Sanchez Munoz , M. De Kock , and P. Forget , “What Is the Place of Clonidine in Anesthesia? Systematic Review and Meta‐Analyses of Randomized Controlled Trials,” Journal of Clinical Anesthesia 38 (2017): 140–153. 28372656 10.1016/j.jclinane.2017.02.003

[16] J. Y. Ju , K. M. Kim , and S. Lee , “Effect of Preoperative Administration of Systemic Alpha‐2 Agonists on Postoperative Pain: A Systematic Review and Meta‐Analysis,” Anesthesia and Pain Medicine 15, no. 2 (2020): 157–166. 33329808 10.17085/apm.2020.15.2.157 PMC7713826

[17] G. Blaudszun , C. Lysakowski , N. Elia , and M. R. Tramèr , “Effect of Perioperative Systemic α2 Agonists on Postoperative Morphine Consumption and Pain Intensity: Systematic Review and Meta‐Analysis of Randomized Controlled Trials,” Anesthesiology 116, no. 6 (2012): 1312–1322. 22546966 10.1097/ALN.0b013e31825681cb

[18] M. Cumpston , T. Li , M. J. Page , et al., “Updated Guidance for Trusted Systematic Reviews: A New Edition of the Cochrane Handbook for Systematic Reviews of Interventions,” Cochrane Database of Systematic Reviews 10, no. 10 (2019): Ed000142. 31643080 10.1002/14651858.ED000142 PMC10284251

[19] D. Moher , A. Liberati , J. Tetzlaff , and D. G. Altman , “Preferred Reporting Items for Systematic Reviews and Meta‐Analyses: The PRISMA Statement,” BMJ (Clinical Research Edition) 339 (2009): b2535. 10.1136/bmj.b2535 PMC2714657 19622551

[20] L. Shamseer , D. Moher , M. Clarke , et al., “Preferred Reporting Items for Systematic Review and Meta‐Analysis Protocols (PRISMA‐P) 2015: Elaboration and Explanation,” BMJ (Clinical Research Edition) 350 (2015): g7647. 10.1136/bmj.g7647 25555855

[21] “Guidance of Good Clinical Practice E6(R2),” 2025, https://www.ema.europa.eu/en/documents/scientific‐guideline/ich‐e6‐r3‐guideline‐good‐clinical‐practice‐gcp‐step‐5_en.pdf.

[22] R. B. Haynes , K. A. McKibbon , N. L. Wilczynski , S. D. Walter , and S. R. Werre , “Optimal Search Strategies for Retrieving Scientifically Strong Studies of Treatment From Medline: Analytical Survey,” BMJ (Clinical Research Edition) 330, no. 7501 (2005): 1179. 10.1136/bmj.38446.498542.8F PMC558012 15894554

[23] J. A. C. Sterne , J. Savović , M. J. Page , et al., “RoB 2: A Revised Tool for Assessing Risk of Bias in Randomised Trials,” BMJ (Clinical Research Edition) 366 (2019): l4898. 10.1136/bmj.l4898 31462531

[24] J. T. J. Higgins , “Cochrane Handbook for Systematic Reviews of Intervensions Version 6.5,” 2024, https://training.cochrane.org/handbook/current.

[25] X. Wan , W. Wang , J. Liu , and T. Tong , “Estimating the Sample Mean and Standard Deviation From the Sample Size, Median, Range and/or Interquartile Range,” BMC Medical Research Methodology 14 (2014): 135. 25524443 10.1186/1471-2288-14-135 PMC4383202

[26] J. P. Higgins and S. G. Thompson , “Quantifying Heterogeneity in a Meta‐Analysis,” Statistics in Medicine 21, no. 11 (2002): 1539–1558. 12111919 10.1002/sim.1186

[27] J. P. Higgins , S. G. Thompson , J. J. Deeks , and D. G. Altman , “Measuring Inconsistency in Meta‐Analyses,” BMJ 327, no. 7414 (2003): 557–560. 12958120 10.1136/bmj.327.7414.557 PMC192859

[28] Cochrane , https://www.cochrane.org/events/identifying‐publication‐bias‐meta‐analyses‐continuous‐outcomes.

[29] A. Dechartres , L. Trinquart , I. Boutron , and P. Ravaud , “Influence of Trial Sample Size on Treatment Effect Estimates: Meta‐Epidemiological Study,” BMJ 346 (2013): f2304. 23616031 10.1136/bmj.f2304 PMC3634626

[30] J. A. Sterne , M. Egger , and G. D. Smith , “Systematic Reviews in Health Care: Investigating and Dealing With Publication and Other Biases in Meta‐Analysis,” BMJ (Clinical Research Edition) 323, no. 7304 (2001): 101–105. 10.1136/bmj.323.7304.101 PMC1120714 11451790

[31] J. C. Jakobsen , J. Wetterslev , P. Winkel , T. Lange , and C. Gluud , “Thresholds for Statistical and Clinical Significance in Systematic Reviews With Meta‐Analytic Methods,” BMC Medical Research Methodology 14 (2014): 120. 25416419 10.1186/1471-2288-14-120 PMC4251848

[32] J. Wetterslev , J. C. Jakobsen , and C. Gluud , “Trial Sequential Analysis in Systematic Reviews With Meta‐Analysis,” BMC Medical Research Methodology 17, no. 1 (2017): 39. 28264661 10.1186/s12874-017-0315-7 PMC5397700

[33] G. H. Guyatt , A. D. Oxman , G. E. Vist , et al., “GRADE: An Emerging Consensus on Rating Quality of Evidence and Strength of Recommendations,” BMJ 336, no. 7650 (2008): 924–926. 18436948 10.1136/bmj.39489.470347.AD PMC2335261

