# Supplementary material for: The Beneficial and Harmful Effects of Perioperative Clonidine: Protocol for a Systematic Review With Meta‐Analysis
Source: Acta Anaesthesiol Scand. 2025 Jul 15;69(7):e70101. doi: 10.1111/aas.70101 (PMC12264455; doi:10.1111/aas.70101)
Supplement: Supplementary file 1 — Data S1. Supporting Information. [file AAS-69-0-s001.pdf]

Search strategies for
Perioperative clonidine for postoperative pain
(S Birkebæk)
Preliminary search strategies prepared 1 July 2025

Total number of records identified records
Number of duplicates removed records
Number of records in final list records

Cochrane Central Register of Controlled Trials (Latest issue) in the Cochrane Library
#1 MeSH descriptor: [Pain, Postoperative] explode all trees
#2 MeSH descriptor: [Pain Management] explode all trees
#3 ((postoperat* or post-operat* or (post next operat*) or postsurg* or post-surg* or (post next surg*) or perioperativ* or peri-operativ* or (peri next operativ*) or ((after or follow*) near/3 (surger* or operation*))) near/4 (pain* or analgesi*))
#4 #1 or #2 or #3
#5 MeSH descriptor: [Clonidine] explode all trees
#6 (clonidin* or clofenil* or klofenil* or clofelin* or klofelin* or clophelin* or isoglaucon* or hemiton* or gemiton* or dixarit* or chlophazolin* or catapres* or kapvay or onyda or duraclon* or nexiclon*)
#7 #5 or #6
#8 #4 and #7

MEDLINE Ovid (1946 to the date of the search)
1. exp Pain, Postoperative/
2. exp Pain Management/
3. ((postoperat* or post-operat* or "post operat*" or postsurg* or post-surg* or "post surg*" or perioperativ* or peri-operativ* or "peri operativ*" or ((after or follow*) adj3 (surger* or operation*))) adj4 (pain* or analgesi*)).mp.
4. 1 or 2 or 3
5. Clonidine/
6. (clonidin* or clofenil* or klofenil* or clofelin* or klofelin* or clophelin* or isoglaucon* or hemiton* or gemiton* or dixarit* or chlophazolin* or catapres* or kapvay or onyda or duraclon* or nexiclon*).mp.
7. 5 or 6
8. 4 and 7
9. (randomized controlled trial or controlled clinical trial or retracted publication or retraction of publication).pt.
10. clinical trials as topic.sh.
11. (random* or placebo*).ab. or trial.ti.
12. 9 or 10 or 11
13. exp animals/ not humans.sh.
14. 12 not 13
15. 8 and 14

Embase Ovid (1974 to the date of the search)
1. exp postoperative pain/
2. exp postoperative analgesia/
3. ((postoperat* or post-operat* or "post operat*" or postsurg* or post-surg* or "post surg*" or perioperativ* or peri-operativ* or "peri operativ*" or ((after or follow*) adj3 (surger* or operation*))) adj4 (pain* or analgesi*)).mp. [mp=title, abstract, heading word, drug trade name, original title, device manufacturer, drug manufacturer, device trade name, keyword heading word, floating subheading word, candidate term word]
4. 1 or 2 or 3
5. exp clonidine/
6. (clonidin* or clofenil* or klofenil* or clofelin* or klofelin* or clophelin* or isoglaucon* or hemiton* or gemiton* or dixarit* or chlophazolin* or catapres* or kapvay or onyda or duraclon* or nexiclon*).mp. [mp=title, abstract, heading word, drug trade name, original title, device manufacturer, drug manufacturer, device trade name, keyword heading word, floating subheading word, candidate term word]
7. 5 or 6
8. 4 and 7
9. Randomized controlled trial/ or Controlled clinical study/ or randomization/ or intermethod comparison/ or double blind procedure/ or human experiment/ or retracted article/
10. (random$ or placebo or parallel group$1 or crossover or cross over or assigned or allocated or volunteer or volunteers).ti,ab.

11. (compare or compared or comparison or trial).ti.
12. ((evaluated or evaluate or evaluating or assessed or assess) and (compare or compared or comparing or comparison)).ab.
13. (open adj label).ti,ab.
14. ((double or single or doubly or singly) adj (blind or blinded or blindly)).ti,ab.
15. ((assign$ or match or matched or allocation) adj5 (alternate or group$1 or intervention$1 or patient$1 or subject$1 or participant$1)).ti,ab.
16. (controlled adj7 (study or design or trial)).ti,ab.
17. (erratum or tombstone).pt. or yes.ne.
18. or/9-17
19. (random$ adj sampl$ adj7 ('cross section$' or questionnaire$ or survey$ or database$1)).ti,ab. not (comparative study/ or controlled study/ or randomi?ed controlled.ti,ab. or randomly assigned.ti,ab.)
20. Cross-sectional study/ not (randomized controlled trial/ or controlled clinical study/ or controlled study/ or randomi?ed controlled.ti,ab. or control group$1.ti,ab.)
21. (((case adj control$) and random$) not randomi?ed controlled).ti,ab.
22. (Systematic review not (trial or study)).ti.
23. (nonrandom$ not random$).ti,ab.
24. 'Random field$'.ti,ab.
25. (random cluster adj3 sampl$).ti,ab.
26. (review.ab. and review.pt.) not trial.ti.
27. 'we searched'.ab. and (review.ti. or review.pt.)
28. 'update review'.ab.
29. (databases adj4 searched).ab.
30. (rat or rats or mouse or mice or swine or porcine or murine or sheep or lambs or pigs or piglets or rabbit or rabbits or cat or cats or dog or dogs or cattle or bovine or monkey or monkeys or trout or marmoset$1).ti. and animal experiment/
31. Animal experiment/ not (human experiment/ or human/)
32. or/19-31
33. 18 not 32
34. 8 and 33

LILACS (VHL Regional Portal; 1982 to the date of the search)
((mh:(pain, postoperative OR c23.550.767.700 OR c23.888.592.612.832 OR pain management OR e02.745 OR n04.590.607.500)) OR (((postoperat* OR post-operat* OR "post operat*" OR postsurg* OR post-surg* OR "post surg*" OR perioperativ* OR peri-operativ* OR "peri operativ*" OR ((after OR follow*) AND (surger* OR operation*))) AND (pain* OR analgesi*)))) AND ((mh:(clonidine OR d03.383.129.308.436.500)) OR ((clonidin* OR clofenil* OR klofenil* OR clofelin* OR klofelin* OR clophelin* OR isoglaucon* OR hemiton* OR gemiton* OR dixarit* OR chlophazolin* OR catapres* OR kapvay OR onyda OR duraclon* OR nexiclon*))) AND db:("LILACS")

Science Citation Index Expanded (1900 to the date of the search) and Conference Proceedings Citation Index – Science (1990 to the date of the search) (Web of Science)
#7 #5 AND #6
#6 TS=(random* or blind* or placebo* or meta-analys* or trial*)
#5 #3 AND #4
#4 TS=(clonidin* or clofenil* or klofenil* or clofelin* or klofelin* or clophelin* or isoglaucon* or hemiton* or gemiton* or dixarit* or chlophazolin* or catapres* or kapvay or onyda or duraclon* or nexiclon*)
#3 #2 AND #1
#2 TS=(pain* or analgesi*)
#1 TS=((postoperat* or post-operat* or (post next operat*) or postsurg* or post-surg* or (post next surg*) or perioperativ* or peri-operativ* or (peri next operativ*) or ((after or follow*) near/3 surger*) or ((after or follow*) near/3 operation*)))
